# Modern contraceptive utilization and associated factors among postpartum women in Kena Woreda, Konso Zone, South Ethiopian Regional State, Ethiopia, 2023: mixed type community based cross-sectional study design

**DOI:** 10.1186/s40834-024-00292-w

**Published:** 2024-06-24

**Authors:** Abdulkerim Hassen Moloro, Solomon Worku Beza, Million Abate Kumsa

**Affiliations:** 1 Department of Nursing, College of Medicine and Health Sciences, Samara University, P.O.Box: 132, Samara, Ethiopia; 2 GAMBY Medical and Business College, Addis Ababa, Ethiopia

**Keywords:** Postpartum modern contraceptive, Postpartum mother, Kena woreda, Konso zone, Ethiopia

## Abstract

**Background:**

Even though family planning 2020 has made remarkable progress about solving the issue of unmet need for family planning, 70% of women in a developing countries who do not want to conceive are not using it. There are limited research that provided detail information regarding barriers of modern contraceptive utilization during postpartum period in the study area. In addition, previous study also recommended that to conduct using mixed quantitative and qualitative design for further investigations to answer these “why” questions and narrow these gaps.

**Objective:**

This study aimed to assess postpartum modern contraceptive utilization and associated factors among postpartum women in Kena woreda, Konso zone, South Ethiopian Regional State, Ethiopia, 2023.

**Methods:**

A mixed type community based cross-sectional study design was conducted among 605 women in Kena woreda, from September 1–30/2023 out of 628 sampled mothers. Multistage sampling technique was used to select study participant and data was collected using semi-structured pretested questionnaire and entered in to Epi data version 3.1 and then exported to STATA version 14 for analysis for quantitative. The association between variables was analyzed using bivariate and multivariable binary logistic regression and level of significant determined with adjusted odd ratio at 95% CI and P-value less than < 0.05. After translation and transcription, manual thematic analysis was applied to the qualitative data.

**Results:**

The prevalence of modern contraceptive use among women during postpartum period in Kena woreda was found to be 39.01% [95% CI: 35.18–42.96%]. Menses resumed (AOR = 1.63; 95% CI: 1.02, 2.59), linked to the family planning unit during their child`s immunization (AOR = 2.17; 95% CI: 1.45, 3.25), family planning counselling during antenatal care visit (AOR = 1.63; 95% CI: 1.10, 2.42) and good knowledge towards modern contraceptive (AOR = 1.53; 95% CI: 1.03, 2.26) were factors associated with postpartum contraceptive utilization. Partner oppose, myths and misconception, need for excess family size, religious prohibition, fear of side effect,menses not resumed, lack of counselling and privacy room, and lack of transportation to health facility were barriers to modern postpartum contraceptive utilization.

**Conclusions and recommendations:**

The utilization of postpartum contraceptives was found to be lower than the target set by the 2020/21 national reproductive health strategy plan, which aimed to increase contraceptive method usage to 50%. Menses resumed, family planning counselling during antenatal care visit, linked to the family planning unit during child immunization and good knowledge were factors associated to modern postpartum contraceptive utilization. Strengthening service integration and family planning counseling during antenatal care visits and encourage mothers to start using modern family planning methods before menses resume are important. Overcoming barriers including partner opposition, myths, religious beliefs, fear of side effects, lack of counseling at health facilities, and transportation challenges is essential.

## Introduction

### Background

Family planning is described as the ability of individuals and couples to achieve their desired number of children in a family when they have children, the age range between children, and the timing of their births by using contraceptive methods [1, 2]. Postpartum modern contraceptive utilization is defined as women who have ever used any type of family planning method or avoidance of closely spaced pregnancies and unintended pregnancy during the first 12 months after she gave birth [3]. Intrauterine contraceptive device (IUD), implants, injectable, pills, and emergency contraception are among the modern contraceptives [4].

Globally, approximately 810 deaths associated with pregnancy and childbirth are recorded daily and about 94% of these maternal deaths occur in low-income and middle-income countries [5]. Sub-Saharan Africa accounts for approximately 66% of maternal death when the majority of their causes are preventable [6]. Ethiopia has a larger share of the World maternal death as 14,000 maternal deaths reported as of the year 2017; hence Ethiopia has vast homework to achieve SDG4 & 5 by 2030 [6]. The magnitude of postpartum modern contraception utilization in Ethiopia ranges from 12.05 to 80.32% [7, 8].

Contraceptive use has reduced maternal death by 40% in the last 25 years worldwide and a further 30% of maternal death would fall if all women who want to avoid pregnancy use an effective contraceptive method in developing countries [9]. Increasing the accessibility of contraception methods among postpartum women is an important strategy because women may initiate sexual activity before obtaining FP methods at their 6-week postpartum visit [10, 11]. The government of Ethiopia is working intensively to ensure affordable and accessible contraceptive methods. The country had written the Health Sector Transformation Plan to reach additional 6.2 million women and increase contraceptive prevalence to 55% until 2020 E.C.

Even though family planning 2020 has made remarkable progress about solving the issue of unmet need for family planning, 70% of women in a developing country who do not want to conceive are not using it [12]. In addition, the Ethiopian demographic health survey (EDHS) 2016 reported that 1in 5 married women had an unmet need for FP [13–15].

According to different cross-sectional-based studies; reproductive health characteristics like resumed menses, resumed sexual intercourse and fertility desire [16], mothers who received antenatal care service [17], women who gave birth with the assistance of a skilled birth attendant [18],maternal age, educational status [19], religious [20], poor economic status [21], and knowledge about contraceptive method [22] were significant factors of postpartum family planning utilization.

As the WHO recommended for better maternal and child health outcomes, postpartum women should wait for an interval of at least 2 years following a live birth before getting pregnant again [23]. In an effort to achieve a better future for all, SDG 3 targeted to reduce the maternal mortality ratio to less than 70 per 100,000 live births by 2030. The SDG plans to ensure universal access to sexual and reproductive health-care services including for family planning [24], which will be measured by the proportion of reproductive age women who have their need for FP satisfied with modern methods of contraception.

In the study area, there is limited research on postpartum contraceptive utilization. Additionally, due to traditional norms, contraceptive use has not been widely accepted. To address this gap, a qualitative study was conducted to explore sociocultural, perceptual, and economic barriers related to the utilization of modern postpartum contraceptives. Depending on previous research, which recommended a mixed quantitative and qualitative approach [25], this study aimed to assess postpartum modern contraceptive utilization and associated factors among women who delivered within the last twelve months in the Kena woreda, Konso zone of the SNNP Region, Ethiopia.

## Methods

### Study area and period

The Kena woreda is located in Konso zone, at 650 Km southwestern of Addis Ababa and found in the South Ethiopia Regional State of Ethiopia. The Kena woreda has a total population of 307,321, of whom 148,070 are men and 159,251 women and 15,048 households, with the average of 5.24 family sizes according to the 2007 national census which was conducted by the Central Statistical Agency of Ethiopia (CSA). The socioeconomic status of the community is characterized by local sectors such as beekeeping, cotton weaving, and agriculture. The Kena woreda divided into 13 kebeles. There are 4 governmental health centers, 19 health post, 3 private clinics and 1 drug store. All government health facilities and private clinics currently providing health services including MCH services in the kena woreda. The study was conducted from September 1–30, 2023 in Kena woreda.

### Study design

A mixed type community-based cross sectional study, supplemented by qualitative was conducted.

#### Source population

All women who gave childbirth within the last twelve months and live in the Kena woreda prior to this study were considered as a source population.

#### Study population for quantitative study

All randomly selected mothers who gave birth in the last twelve months, in the selected kebeles of Kena woreda were considered as a study population.

#### Study population for qualitative study

All purposively selected mothers with history of postnatal contraceptive use and non-users, health extension workers (HEWs), husbands of family planning user and non-user, Maternal and child health coordinators and health professionals who work family planning service were considered as a study population for qualitative study.

#### Inclusion criteria

All women’s who give birth in the last 12 months period before data collection regardless of their birth outcome were included in the study.

#### Exclusion criteria

Woman who lived in the study area for less than 6 months.

Women who cannot communicate and critically ill.

#### Exclusion criteria for qualitative part

Participants interviewed in the quantitative survey were excluded from in-depth interview.

### Sample size determination

#### Sample size determination for both quantitative and qualitative

Sample size for both first objective and second objective were calculated using ***Open Epi online software version 3.0.1*** to use large sample size. Accordingly sample size for first objective was calculated using single population proportion formula considering the following assumptions: 54.7% prevalence of postpartum family planning utilization from study conducted in Addis Zemen town, South Gondar, Ethiopia in 2019 [25], 5% margin of error and 95% CI and and finaly ***381*** obtained. Since it is multi stage sampling technique used, design effect applied and multiplied by 1.5 becomes 571 and by adding a 10% non-response rate, the first objective sample size becomes **628.**

Qualitative data were collected using In-depth interviews among postnatal mothers who use and not use contraceptive, husband and from key informants. The number of sample was determined using the principle of “saturation”- women and key informants were asked to participate in interviews until additional interviews did not provide additional evidence about the main themes of interest and a total of 13 (2 modern contraceptive users mother, 6 non-users, 1 husband, 4 health care provider 2 health extension workers, and 2 maternal and child health care coordinator) participants included in In-depth interviews.

#### Sampling procedure for quantitative and qualitative study

**Fig. 1:**
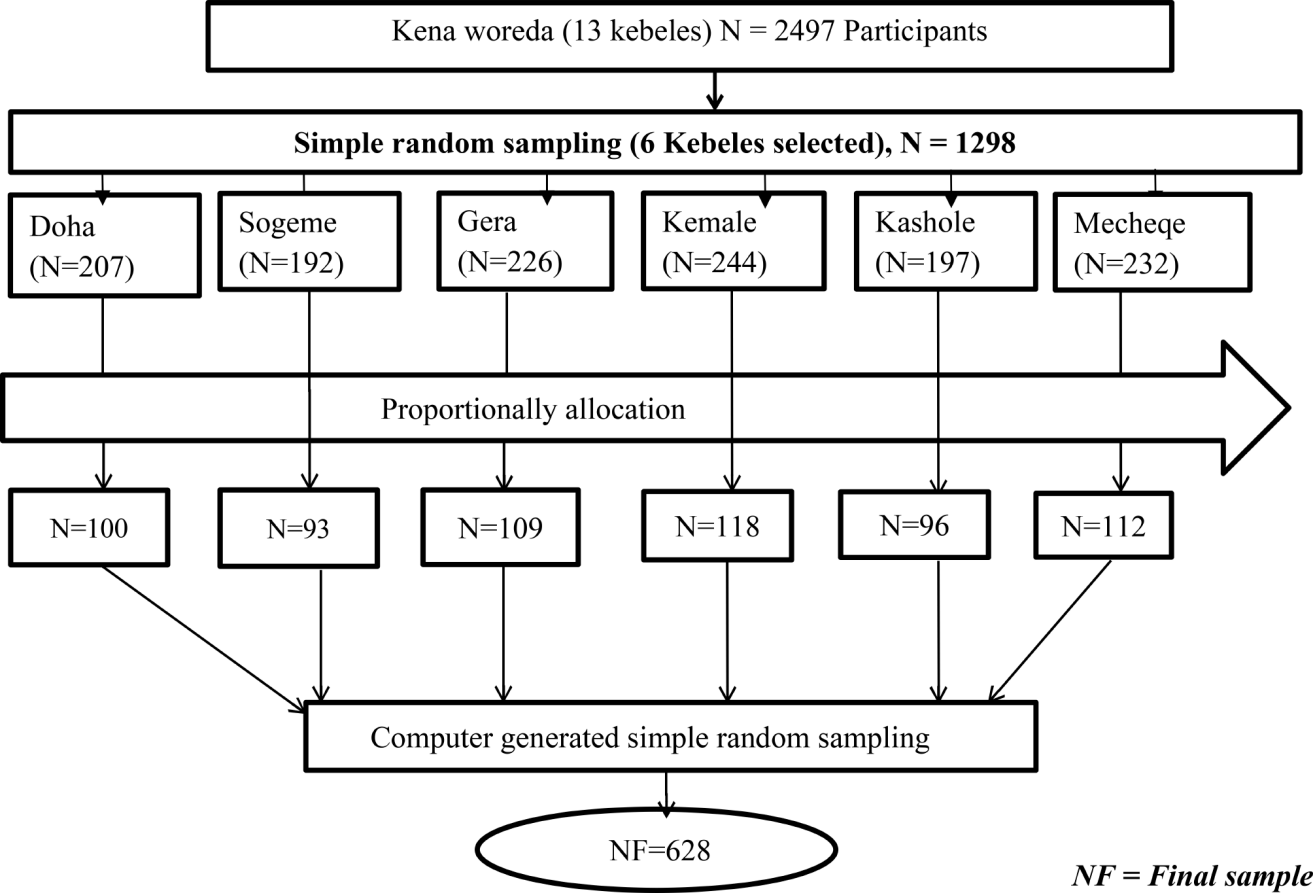
Schematic representation of sampling techinque to assess postpartum modern contraceptive utilization among postpartum women who gave birth in the last 12 months in Kena woreda, Konso zone, South Ethiopia Regional State of Ethiopia, 2023

A multistage sampling technique was used to select the study participants. In the six kebeles, there were 1298 women who gave childbirth in the last twelve months in the six kebeles. First, six representative kebeles were selected from thirteen kebeles using simple random sampling technique (lottery methods). In the second stage, computer generated sampling method was used to select the households from six selected kebeles. The sample frame of households (mothers who delivered in the last one year) was prepared in all selected kebeles from mother’s registration book which was found from health extension workers. Then study participants were proportionally allocated for each selected kebeles (See figure 1).

Finally, the calculated 628 study participants were selected through computer generated random sampling technique. One mother was selected per household. If two or more eligible women were encountered in one household, only one was included using lottery method and if no eligible women are identified in the selected household, the next household nearest to eligible household located in a clockwise direction was visited until the desired sample size were achieved. If the participant in the selected household was not present at the time of data collection, three revisits were made to interview the woman. The next household replaced for those absent household after tree revisit.

For qualitative data, the heterogeneous (maximum variation) type of purposive sampling technique was employed to select the participants until data saturation were reached. Modern contraceptive method users and non-users, husbands, health extension workers, maternal and child health care coordinator, and health professionals work on family planning service were included in the qualitative study. All participants were selected purposively based on their close relationship with mother and involvement in the community. The numbers of in-depth interview were determined based on level of saturation of the required information. Thirteen participants reached saturation, and a total of 13 participants were included for qualitative data.

#### Dependent variables

- Postpartum modern contraceptive utilization.

#### Independent variables

- ***Socio demographic variables***: Age, marital status, educational status, religion, ethnicity, occupational status of the mother, and husband educational status and occupation,.
- ***Knowledge***: Heard any methods, type of contraceptive, source of information, time of contraceptive start and benefit of family planning.
- ***Reproductive and maternal health service use-related characteristics***: Parity, birth interval, pregnancy status, discuss about family planning with partner/husband, ANC care, ANC Family planning counseling, postnatal care, postnatal FP counselling, place of delivery, delivery assistant, immunization visit for child, reproductive intention, menstrual resumption, sexual resumption, and postpartum period in weeks.

### Operational and term definitions

- ***Modern contraceptives***: Sterilization (male and female), intrauterine devices, implants, oral contraceptives, condoms (male and female), injectable, emergency contraceptive pills and spermicidal agents [26].
- ***Postpartum contraceptive utilization***: Utilization of the postpartum family planning was defined as a postpartum woman using any one of modern postpartum FP methods (progesterone-only pills, intrauterine contraceptive device, injectables, dual method, sterilisation (permanent FP method) or implants) during the first 12 months after she gave birth [27–29]. The utilization was measured by mothers’ words by yes or no options for use (yes = 1, no = 0).
- **Knowledge of postpartum contraceptive**: Mother was considered to have good knowledge if she correctly answered greater than or equal to the mean score of the total knowledge assessing questions and considered as poor knowledge if she answered less than to the mean score of the total knowledge assessing questions [8, 30, 31].

### Data collection tool and procedure

#### Data collection tool and procedure for quantitative and qualitative

The quantitative data were collected via interviewer administered approach by using semi-structured and pretested questionnaires adapted from different literatures [8, 16, 20, 25, 28, 31, 32]. The tool first prepared in English language after reviewing different literature. The data were collected by trained 5 Diploma Nurse in which 4 are data collectors and 1 is supervisors from selected Keble’s. Training was given for data collectors and supervisors for one day on data collection procedures, interview techniques and confidentiality of the information obtained from the respondents. Finally, data collectors were interviewed participants face to face at home for 25–30 min.

The qualitative data were collected using in-depth interviews by two individuals who have previous experience until information saturation was reached. Audio record and note were taken for each interview, after obtaining voluntary written or oral signed consent from the interview participants.

### Data quality control

#### Data quality control for quantitative and qualitative

Data quality was ensured during collection, entry and analysis. Before conducting the main study, pretest was carried out on 5% [31] of the sample size in Fasha kebele which was non-selected kebele and necessary modification was made. The principal investigator and supervisors were conducted a day-to-day on-site supervision during the whole period of data collection. At the end of each day, the questionnaires were reviewed and checked for completeness and accuracy by the supervisors and investigator. Then corrective modification was made by the principal investigators.

To maintain validity, the questionnaire was prepared in English and then translated to local language (Konso language version) and back translated to English by language experts to ensure consistency and accuracy. Data were checked for completeness, accuracy, clarity, and consistency before being entered into the software. Proper coding and categorization of data was maintained for the quality of the data to be analyzed.

Open ended in-depth interview questions taken from different literatures were used for qualitative data collection. The data was collected by the principal investigator by audio recording and note taking. The accuracy for the qualitative component was addressed by ensuring credibility, dependability and transferability. Furthermore, sufficient time (30–40 min) objective and impartial view maintained to collect data were added the reliability of the data.

#### Data Processing and Analysis for quantitative and qualitative

The pre coded data were entered into Epi data version 3.1software and exported to STATA version 14 software for statistical analysis. Data exploration, editing and cleaning were undertaken before analysis. Descriptive statistics such as mean and standard deviation were used for continuous data and percentage, frequency, tables and cross tabulation were used for categorical data.

Regarding multi-collinearity among independent variables, STATA itself was controlled and checked via variance inflation factor (VIF) and tolerance. Variable VIF greater than 10 and tolerance less than 0.1 were removed in STATA. Binary logistic regression analysis was conducted to see factor associated with modern contraceptive use and variables with P value less than 0.25 in bivariable analysis will be transferred for multivariable logistic regression to control the confounders.

Final model fitness were checked by Hosmer and Lemeshow test and model adequacy was declared when p-value > 0.05. Accordingly, final model of Hosmer and Lemeshow showed 0.098. The significance was checked and declared using p-value less than 0.05 and 95% confidence interval in the final model. Strength of association was interpreted by using adjusted odds ratio with 95% confidence interval.

Primarily, audio recorded data were heard repeatedly until the principal investigator became intimately familiar with the contents. The audio taped qualitative data were first transcribed in to original language and then translated to English language by principal investigator. Then, codes or terms were identified and tallied to come up with same categories, which later used to establish themes based on the objective of the study. Unique concepts were identified and trials were made to elaborate more and reported on the final result. Finally, thematic analysis was done manually and the findings were triangulated with the quantitative one.

##### Ethical consideration

Ethical approval was obtained from the Institutional Ethical Review committee (IERC) of GAMBY Medical and Business College with IERC protocol number (Ref.No_GMBCIB/IRB/846/2023). Following the approval by IERC, official letter of co-operation were written to Kena woreda health administration office and in turn the woreda health administration office was wrote letters to each selected kebeles and village’s administration office in order to get permission and cooperation. The oral informed consent from the respondent was obtained after the purpose and objective of study explained. To ensure confidentiality, a name of respondents was replaced by code numbers. Participants were given the chance to ask any doubt about the study and made free to refuse or stop the interview at any moment they want.

## Results

### Socio-demographic characteristics

Out of the 628 respondents sampled, six hundred five women participated in the study, resulting in a response rate of 96.34%. The mean age of the mothers was 28.28 (SD ± 4.91), ranging from 17 to 39 years. Majority of the respondents 286(47.27%) were followers of the protestant religion and 476 (78.68%) of the study participants belong to the Konso ethnicity. In this study, 564 (93.22%) of the mothers were married and 440 (23.33%) were housewives. Of the mothers, 419 (69.26%) were unable to read and write followed by primary education level which represented, 71(11.74%)(See Table 1).

**Table 1:** Socio-demographic characteristics of women in postpartum period in Kena woreda, Konso zone South Ethiopia Regional State of Ethiopia, 2023 (*n* = 605)

Variables	Category	Frequency	Percent (%)
Age of the mother	15–24	124	20.5
	25–34	403	66.6
	≥ 35	78	12.9
Marital status	Single	30	4.9
	Married	564	93.3
	Divorced	9	1.5
	Widowed	2	0.3
Religion	Orthodox	278	45.9
	Protestant	286	47.3
	Muslim	41	6.8
Ethnicity	Konso	476	78.7
	Gawwada	51	8.4
	Oromo	49	8.1
	Amhara	29	4.8
Occupation of the mother	House wife	440	72.9
	Self-employees	57	9.4
	Government employees	47	7.8
	Merchants	34	5.6
	Labor work	11	1.8
	Others*	15	2.5
Educational status	Unable to read and write	419	69.3
	Primary education	71	11.7
	Secondary education	66	10.9
	Diploma and above	49	8.1

Others* = Farming, job less

### Reproductive and maternal health characteristics

Mothers had a mean parity of 2.75 (SD ± 1.56), and the mean value of living children was 2.71 (SD ± 1.55). In terms of their reproductive intention, 210 (34.71%) mothers desire to have children, while 197 (32.56%) desire to space out their children over time. More over half of the mothers, 349 (57.69%), stated that their periods had returned after giving birth, and more than three-quarters, 442(73.06%), reported that they had resumed their sexual activities. Almost all mothers, 559(92.4%) had ANC visit for last pregnancy and 241(43.11%) had four and above times ANC visit.

Regarding family planning counselling, more than half of the mothers, 298(53.31%) was counselled during ANC visit and 180(29.75%) mothers were counselled during postnatal care visit. 390(64.46%) of the mothers, had delivered at health institution and 215(35.54%) of the mothers had assisted by midwifes during delivery. Majority of respondents, 543 (89.75%) had visit immunization clinic for child and 177(32.6%) had linked to family planning unit during child immunization. (See Table 2).

**Table 2:** Reproductive and maternal health characteristics of women in postpartum period in Kena woreda, Konso zone, South Ethiopia Regional State of Ethiopia, 2023 (*n* = 605)

Variables	Category	Frequency	Percent (%)
Parity	1–2	333	55
	3–4	184	30.5
	≥ 5	88	14.5
Number of live children	1–2	333	55
	3–4	192	31.8
	≥ 5	80	13.2
Pregnancy condition	Planned	493	81.5
	Unplanned	112	18.5
Birth interval in month	< 24	364	75.7
	24–48	72	14.9
	≥ 48	45	9.4
Discuss with husband about FP	Yes	388	64.1
	No	217	35.9
ANC visit for last pregnancy	Yes	559	92.4
	No	46	7.6
If yes, how many times attend ANC	One visit	73	13
	Two visit	101	18
	Three visit	144	25.9
	Four and above	241	43.1
ANC Family Planning Counselling?	Yes	298	53.3
	No	261	46.7
Post-natal care visit	No	425	70.3
	Yes	180	29.7
Postnatal FP counselling	No	425	70.4
	Yes	180	29.6
Place of delivery	Health institution	390	64.6
	At home	215	35.4
Delivery assistant	Midwives	231	38.2
	Doctors	55	9
	Nurses	104	17.3
	Untrained traditional birth attendant	215	35.5
Visit immunization for your child?	Yes	543	89.7
	No	62	10.3
Linked to FP unit during immunization?	No	366	67.4
	Yes	177	32.6
Reproductive intention	Want to space	197	32.6
	Want to limit	83	13.7
	Undecided	115	19
	Want to have child	210	34.7
Who decide the reproductive intention	Husband	57	9.4
	Wife	206	34.1
	Both	341	56.5
Menses resumed	No	256	42.3
	Yes	349	57.7
Resumed sexual activity	No	163	26.9
	Yes	442	73.1
Postpartum period in weeks	0–12	166	27.5
	13–26	245	40.5
	27–38	142	23.5
	39–50	52	8.5

### Knowledge on postpartum modern contraception use

More than three-fourth of the study participants, 467 (77.19%) had heard at least one modern contraceptive method. Majority of the mothers, 440(94.22%) heard about injectable FP methods and 471 (77.85%) responded that postpartum family planning should be started to use after two months of delivery. Majority of the respondents, 536 (88.6%) said about the benefit of postpartum family planning methods utilization is to limit the number of children. Regarding the overall knowledge on postpartum family planning, majority of respondents, 353 (58.35%) had good knowledge of PPFP methods (*See* Table 3).

**Table 3:** Knowledge of modern postpartum family planning methods of women in postpartum period in Kena woreda, Konso zone, South Ethiopia Regional State of Ethiopia, 2023 (*n* = 605)

Variables	Category	Frequency	Percent (%)
**Heard about any modern method of contraception**	Yes	467	77.2
	No	138	22.8
**Which method(s) did you know? (Multiple response)**	Pill(Oral contraceptive)	427	91.4
	Intrauterine Contraceptive Device	225	48.2
	Male condoms	358	76.7
	Female condoms	198	42.4
	Implants	349	74.7
	Injectable contraception (DMPA)	440	94.2
	Emergency hormonal contraception	302	64.7
	Tubal ligation	188	40.3
	Vasectomy	137	29.3
**Source of information**	Health professional	393	84.2
	TV	89	19.1
	Radio	129	27.6
	Friends	106	22.7
	From pamphlets/ booklet/posters	76	16.1
**Timing of starting to use PPFP methods**	Within two months of delivery including immediate	134	22.1
	After two months of delivery	471	77.9
**Benefit of postpartum family planning methods utilization**	Birth spacing	438	72.4
	Limiting the number of children	536	88.6
	Prevention of unwanted pregnancy	412	68.1
	Prevent possible maternal death	519	85.8
	Prevent STIs	459	75.9
**Knowledge of modern FP methods**	Good Knowledge	353	58.4
	Poor Knowledge	252	41.6

### Modern contraceptive use in the postpartum period

**Fig. 2:**
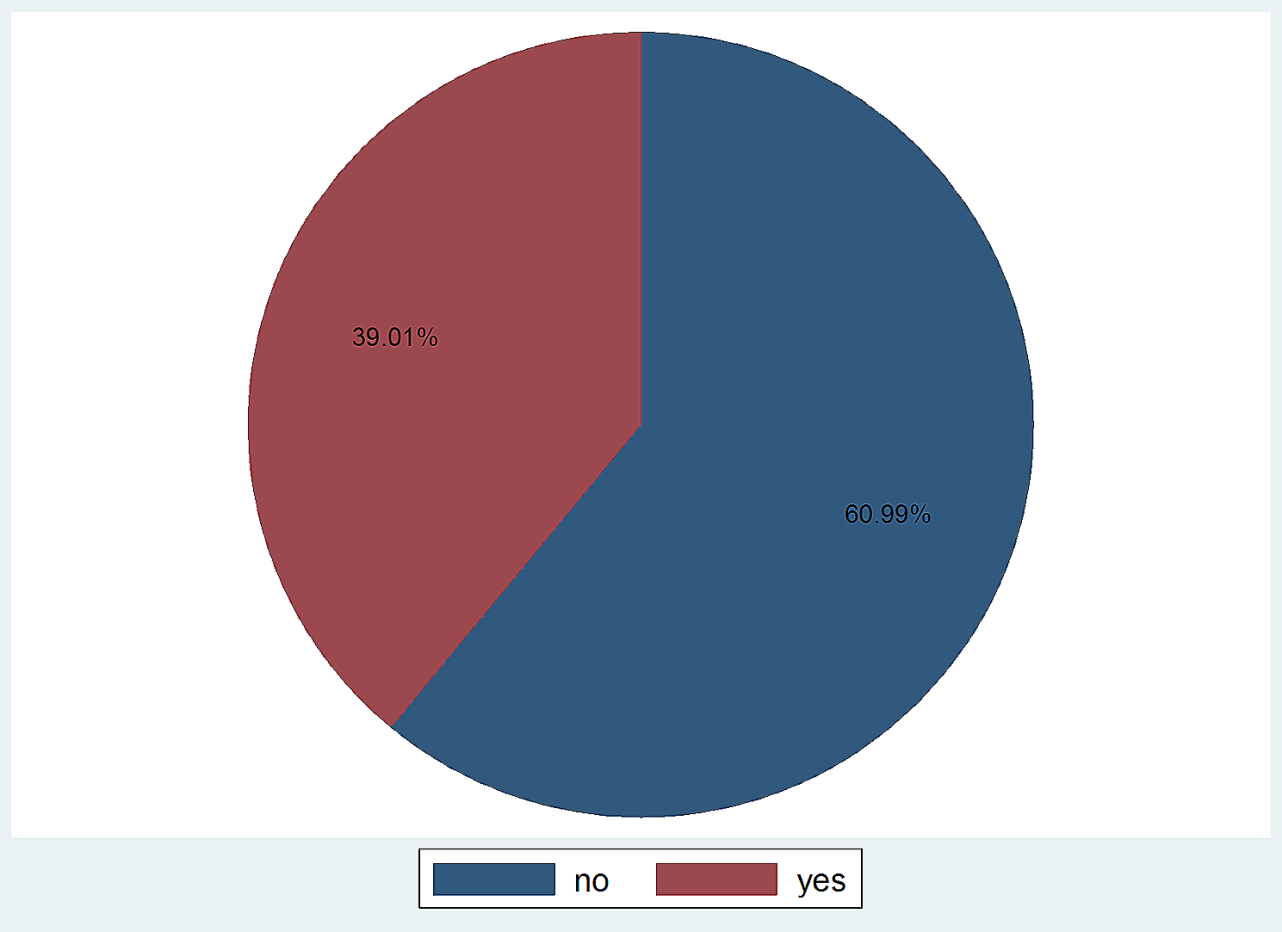
Modern contraceptive use of women in postpartum period in Kena woreda, Konso zone, South Ethiopia Regional State of Ethiopia, 2023 (*n* = 605)

**Fig. 3:**
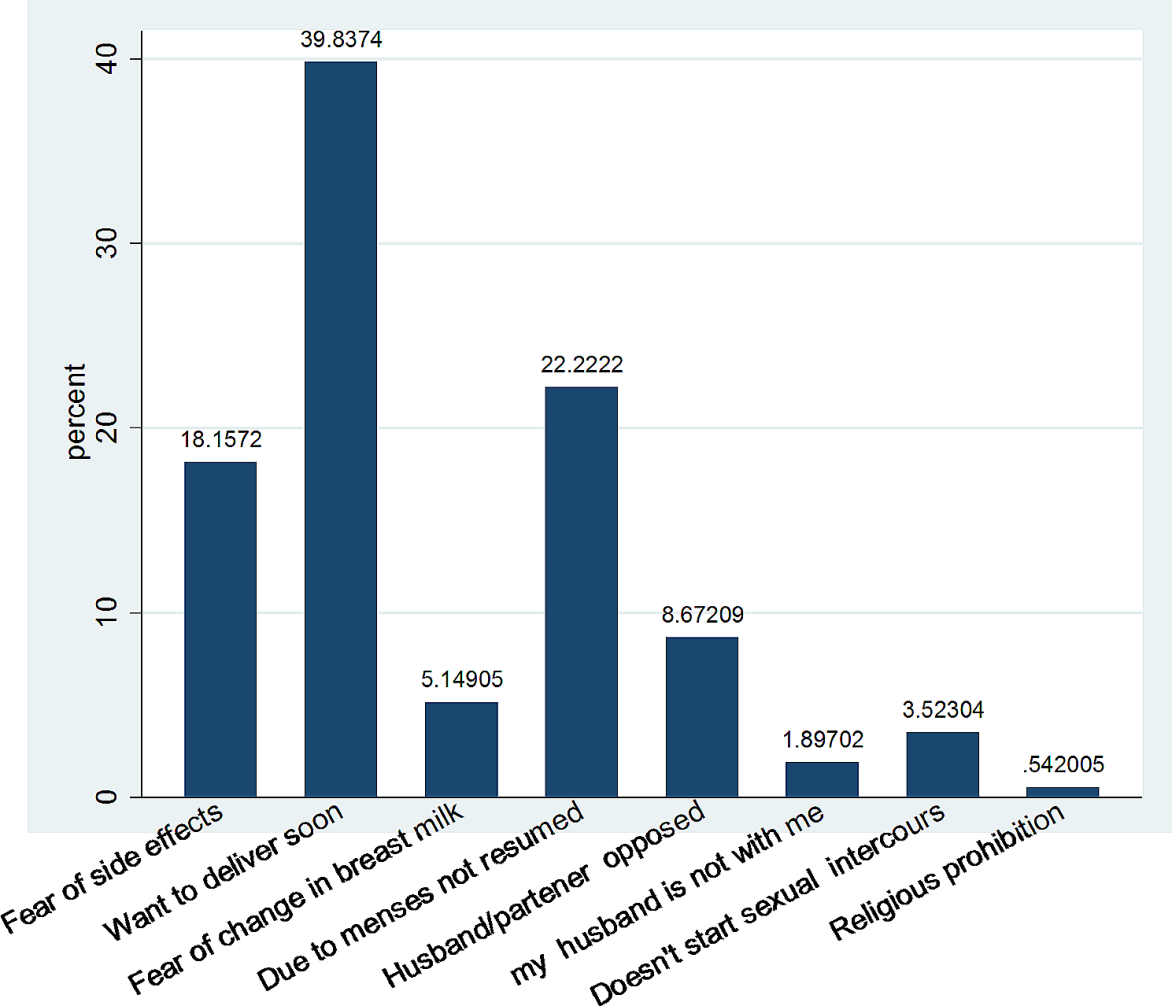
Reasons for not using modern contraceptive during the postpartum period among women in Kena woreda, Konso zone, South Ethiopia Regional State of Ethiopia, 2023 (*n* = 605)

In this study, the prevalence of modern contraceptive use among women in the postpartum period was found to be 236 (39.01%)[95% CI: 35.18–42.96%](*See* Fig. 2). The most commonly used contraceptive method was injectable 118 (50%) followed by implants 89 (37.71%) and pills method users 26(11.02%). One hundred fifty nine (67.37%) of the mothers, who currently use FP methods started using the methods following menstruation, 40 (16.95%) of postpartum mothers began using before menstruation resumed, and 37 (15.68%) of women began using it immediately after giving birth. The majority of mothers, 189 (80.08%) receive PPFP via the public health system, followed by private health facilities 37 (15.68%) and pharmacies/drug sellers 10 (4.24%). The reasons for not using contraceptives during postpartum period for 147 (39.84%) of the participants was feeling of want to deliver soon. Next to this, others 82(22.22%) reported that due to menses not resumed (*See* Fig. 3).

### Factors associated with postpartum modern contraceptive use

Based on bivariable logistic regression, mothers age, ANC visit, ANC counselling about family planning, visit immunization clinic for child, linked to family planning during child’s immunization, menses resumed, resumed sexual activity, postpartum period in weeks, and good knowledge towards modern contraceptives were factors have p-value less than 0.25 and all were transferred to multivariable logistic regression analysis to control the effect of confounder.

Factors which have p-value greater than or equal to 0.25 in bivariable logistic regression for all categories were not considered for multivariable logistic regression analysis. However, in the multivariable logistic regression analysis, mothers aged 15–24, mothers linked to family planning from immunization clinic, postpartum period from 39 to 50 weeks, and good knowledge were identified as predictors of postpartum modern contraceptive use among mothers (*p* < 0.05). Due to multicollinearity STATA itself ommitted the variable named "visit immunization clinic" in the multivariable reggression. For bivariable analysis both confidence interval and p-values were presented but for multivariable analysis only confidence intervals were presented.

Accordingly, findings from multivariable logistic regression showed that, odds of using modern contraceptive during postpartum period among menses resumed mothers were 1.63 more likely than their counterpart (AOR = 1.63; 95% CI: 1.02,2.59). Similarly, odds of mothers who are linked to the FP unit during their child`s immunization were 2.17 more likely to use modern contraceptive method during postpartum period than their counterpart (AOR = 2.17; 95% CI: 1.45, 3.25). Again this study showed that the odds of using modern contraceptive methods during postpartum period among mother counselled about family planning during ANC visit were 1.63 more likely than their counterparts (AOR = 1.63; 95% CI: 1.10, 2.42). Furthermore,  mothers who have good knowledge towards modern contraceptive were 1.53 more likely to use modern contraceptive method during postpartum period than their counterpart (AOR = 1.53; 95% CI: 1.03, 2.26)(*See* Table 4).

**Table 4:** Bivariable and multivariable analysis of factors associated with the use of modern contraceptives during the postpartum period among mothers in Kena woreda, Konso zone, Southern nation and nationalities people of Ethiopia, 2023 (*n* = 605)

Variables	Category	Current use of PPFP		COR(95%CI)	*P*-value	AOR(95%CI)
		Yes	No			
**ANC Visit**	One visit	21(28.8)	52(71.2)	1		1
	Two visit	35(34.7)	66(65.3)	1.31(0.68,2.51)	0.41	1.56(0.74,3.28)
	Three visit	63(43.7)	81(56.3)	1.92(1.05,3.52)	0.034*	1.84(0.93,3.65)
	Four and above	102(42.3)	139(57.7)	1.81(1.03,3.20)	0.039*	1.11(0.58,2.14)
**ANC FP Counselling**	Yes	140(46.9)	158(53.1)	1.96(1.39,2.78)	0.000*	1.63(1.10,2.42)**
	No	81(31.1)	180(68.9)	1		1
**Visit immunization clinic**	Yes	220(40.5)	323(59.5)	1.95(1.08,3.54)	0.027*	***Omitted***
	No	16(25.8)	46(74.2)	1		1
**Linked to FP unit**	Yes	96(54.3)	81(45.7)	2.28(1.58,3.29)	0.000*	2.17(1.45,3.25)**
	No	125(34.2)	241(65.8)	1		1
**Menses resumed**	Yes	163(46.7)	186(53.3)	2.19(1.55,3.09)	0.000*	1.63(1.02,2.59)**
	No	73(28.5)	183(71.5)	1		1
**Resumed sexual activity**	Yes	188(42.5)	254(57.5)	1.77(1.2,2.6)	0.004*	1.56(0.95,2.56)
	No	48(29.5)	115(70.5)	1		1
**Postpartum category in weeks**	0–12	44(26.5)	122(73.5)	1		1
	13–26	97(39.6)	148(60.4)	1.81(1.18,2.79)	0.006*	1.41(0.83,3.40)
	27–38	65(45.7)	77(54.3)	2.34(1.45,3.77)	0.000*	1.46(0.76,2.81)
	39–50	30(57.7)	22(42.3)	3.78(1.97,7.23)	0.000*	2.14(0.93,4.91)
**Knowledge**	Good knowledge	154(43.6)	199(56.4)	1.60(1.14,2.24)	0.006*	1.53(1.03,2.26)**
	Poor knowledge	82(32.5)	170(67.5)	1		1

### Qualitative findings

In-depth interview was conducted among 13 participants (2 health extension workers, 4 non-contraceptive user women, 2 husbands, 2 women who have previous history of contraceptive use, 1 midwifery women who work in ANC at health center and 2 male who coordinate and work at maternal and child health center) to explore the barriers of postpartum contraceptive use. The study saturation was obtained within thirteen participants. To triangulate the findings, in-depth and key informant interviews findings were summarized into themes that emerged during interviews. Generally, most repeatedly mentioned barriers by the participants were categorized in to three main themes (sociocultural barriers, individual barriers and health service related barriers) which included eight sub themes (categories) in it.

### Sociocultural barriers

The sociocultural barriers include four sub-themes: husband/partner oppose, myths and misconception on contraceptive, need for excess family size, and religious prohibition.

### Sub-theme: Husband/partner oppose

During in-depth interviews, nearly all participants consistently highlighted that opposition from husbands serves as a significant barrier to utilizing postpartum contraceptive methods. According to their ideas, women seeking to use contraception must first obtain their husband’s permission. Additionally, they emphasized that women who practice family planning without their husband’s awareness may face serious consequences, including warnings, divorce, physical violence, and family conflicts.

One postpartum mother, 30 years old, expressed her feeling to space her pregnancies by three years due to a previous one-year interval. However, her husband opposed this plan. She stated, ‘*My pregnancies are always close together, and I don’t want to be pregnant again until our child is three years old. Unfortunately, my husband doesn’t allow me to use any modern contraceptive methods*” ***IDI participant (Postpartum contraceptive non-user)***.

During the interviews, a 25-year-old postpartum mother shared her perspective. She mentioned that she currently has an 8-month-old child and prefers not to become pregnant again for the next three years. However, her husband disagrees. Her statements are stated in the following quotes: ‘…*my child is now 8 months old, and I don’t want to conceive until my child is 3 years old, but my husband doesn’t allow me to seek contraceptive services at health facilities*” ***IDI participant (Pospartum contraceptive non user)***.

Furthermore, one postpartum woman, aged 22, reported that she had previously used the injectable contraceptive (Depo Provera). However, her husband expressed opposition, leading her to discontinue its use. She supported her statement with the following quote: ‘*I have used Depo Provera three times in the past, but I stopped after my husband recognized it and warned me to discontinue*” ***IDI participant (Postpartum contraceptive user)***.

### Sub-theme: myths and misconception on contraceptive

During our interviews, many participants emphasized a prevailing belief in the community that modern contraceptive use is contingent upon having a balanced diet. If a husband cannot provide sufficient food, it is believed that contraceptive use may lead to illness and negative health outcomes. Consequently, women who lack access to a balanced diet are discouraged from using contraceptives. For instance, a 31-year-old postpartum mother expressed her desire to use contraceptive methods but hesitated due to the perceived dietary requirements. Her partner, who had used contraceptives before, informed her about this, and her husband also cautioned against using them without an adequate and balanced diet. She evidenced her idea in the following quote “………*I want to use contraceptive methods, but I fear it due to it needs a balanced diet as informed from my partner who used it previously. In addition, my husband also told me that not to use without enough and balanced diet”.****IDI women (postpartum mothers who are non-users of contraceptives).***

Among the community, there is a misconception that contraceptive use prevents future pregnancies and leads to infertility after discontinuation. During a key informant interview, a 32-year-old health care provider shared her ideas in the following quote “….*most of the mothers believe that they cannot become pregnant once they have used and removed contraceptives. Additionally, they perceive that contraceptives cause infertility*”***IDI key infromant participants (Health care provider).***

### Sub-theme: need for excess family size

Another sociocultural factors act as barriers to contraceptive use. One such factor is the cultural desirability of having excess children and maintaining a large family size. Participants consistently reported that their communities associate having many children with love and respect. Consequently, families strive for a larger family size to gain esteem within their clans and society. As a result, husbands and other family members often discourage women from using contraceptive methods, believing that having more children enhances their status. For instance, a 36-year-old husband in an interview stated, “*Our culture encourages us to have excess children to earn love and respect within our clan and society. Additionally, we seek marital alliances with families that have large family sizes, as we believe that if a mother can bear many children, her daughters will also do the same*”. (***IDI participants, Husband of the non-user mother***)

### Sub-theme: religious prohibition

Another’s barriers reported by participants was religious prohibition. Some community members perceive contraceptive use as tantamount to ending a life that has already been conceived, and thus consider it a sin. They hold the belief that God provides for our children and us. A 27-year-old non-user mother expressed her perspective: “*I am still having children, and I want to continue having them without gaps. God (Egzaber) sustains all of us, including our children, and shapes our destinies in terms of what we eat and how we live. Therefore, contraceptive utilization is deemed sinful”****IDI participants (postpartum non-user mother)***.

### Individual barriers

The individual barriers include two sub-themes; fear of side effect, and menses not resumed.

### Sub-theme: fear of side effect

The majority of respondents acknowledged that fear of side effects is a significant barrier to the low utilization of postpartum contraceptives. A 28-year-old non-user mother mentioned that she had heard about side effects such as bleeding and headaches from her friend. Based on her friend’s experience, she was hesitant to use contraceptives herself. The participant’s quote reflects this concern: “….*I want to use contraceptive methods, but I fear side effects like excessive bleeding, dizziness, and headaches, which my friend experienced. Additionally, my friend stopped using contraceptives and advised me not to use them either*” ***IDI participant (Non-user postpartum mother)***.

In addition, another 28-year-old mother stated in the following quotes, *“……I am aware that my friend used the injectable contraceptive (Depo-Provera) in the past, and she found it quite challenging. She mentioned experiencing loss of appetite and significant weight loss.’****IDI participant (Non-user postpartum mother)***.

Furthermore, another 20-year-old mother shared, *‘……I’ve been using the injectable contraceptive Depo-Provera for two years, and I’ve faced some challenges. These include loss of appetite, bleeding, and significant weight loss. If I completed the schedule, I will remove it” ’****IDI participant (Postpartum contraceptive user mother)***.

### Sub-theme: Menses not resumed

Another frequently reported factor was the lack of resumed menstruation. A 34-year-old non-user mother expressed that she did not consider using contraceptives. She justified her decision by saying, ‘…since my menstruation has not resumed, I don’t want to use it” ***IDI women (postpartum non-user mother)***.

### Health service related barriers

The health service related barriers include two sub-themes; lack of counselling and privacy room, lack of accessibility to contraceptive methods and lack of transportation to health facility.

### Sub-theme: lack of counselling and privacy room

According to feedback from in-depth interview participants, the lack of counseling poses a significant barrier to the utilization of postpartum contraception. A 26-year-old healthcare provider highlighted the challenge of insufficient counseling for mothers who deliver in our health facilities due to the absence of a dedicated family planning counseling room. Additionally, she mentioned that counseling and implanon procedures are conducted in the delivery room. Her sample quotes reinforce the idea that “…….*despite efforts to provide counseling, patient overcrowding and busy schedules hinder comprehensive postpartum counseling. As a result, counseling is sometimes delivered at the bedside, during delivery, and during antenatal care (ANC) visits*” ***IDI key informant participants (Health care provider.***

A 29-year-old healthcare provider mentioned that “…….*they provide counseling to mothers who come for child immunization. They observed that mothers are more likely to use postpartum contraceptives when they receive counseling. The provider believes that if both the husband and mother receive counseling simultaneously, they would be more likely to use contraceptives*” ***IDI Key informant participants (Health care provider)***.

### Sub-theme: lack of accessibility to contraceptive methods

Many respondents emphasized that lack of transportation access, poor road infrastructure, and long distances to health facilities contributed to low contraceptive use. Health care providers and maternal and child health coordinators noted that most mothers preferred using Implanon and injectable contraceptives (such as Dipo Provera). However, shortages of these contraceptives sometimes prevented their use. During an interview a 32-year-old health care provider highlighted that “……*mothers often complained about transportation challenges due to distance from health facilities. As a result, they faced difficulties attending antenatal and postnatal care. The shortage of specific contraceptive methods further compounded the issue*” ***IDI Key informant participants (Health care provider).***

## Discussion

In this study the prevalence of modern contraceptive use among women in the postpartum period was found to be 39.01% [95% CI: 35.18–42.96%]. This finding is comparatively in line with other studies conducted in Ethiopia, rural Tigray region 38.3% [33], Oromia regional state 40.7% [34], Hawassa Town 38.6% [35] Durame Town 36.7% [36] and Debre Berhan 41.6% [16]. The findings also in line with studies conducted in Nigeria 39.8% [37], low-income countries of SSA was 37.41% and Eastern Africa 41.36% [38] and Nepal 37% [39].

However, the finding is comparatively lower than to previous studies conducted in different parts of Ethiopia namely, south Gondar 54.7% [25], Debre Tabor town 63% [40], Northwest Ethiopia 60.6% [32], Gozamen district 46.7% [41], Bahir Dar city 48.8% [42], Gondar 45.8% [30], Arba Minch town 44% [43], Aksum 48% [28], Gida Ayana district Oromia region 45.4% [44], Addis Ababa 80.3% [7], Ganta-Afeshum District Eastern Tigray region 68.1% [45], Butajira 47% [46], Dessie Town 44% [17], and Addis Zemen 44% [28]. The possible discrepancy for this prevalence may be justified by the fact that the study area is might be attributed to low maternal health care service utilization. This study was also conducted mainly in rural kebeles where low educational status, poor counselling about contraceptive and lack of transportation to health facilities might be a reason for less utilization of PPFP compared to studies mentioned above as most of them were conducted in the town [47, 48]. Therefore addressing disparities in contraceptive utilization requires a multifaceted approach that considers regional context, education and counselling, and access to healthcare services. By promoting modern contraceptive methods, maternal and child health outcomes can also be improved while advancing human rights and sustainable development [49].

This finding also found to be lower compared with studies conducted in Rural Kenya 86.3% [50], Mexico 47% [51], Kenya 46% [52], Tanzania 46% [53], Ntchisi district hospital Malawi 75% [22], and Rwanda 51% [54]. The possible reason for this discrepancy might be attributed to the differences in policy, variation in service accessibility, and maternal health care service utilization [55].

This finding is also comparatively greater than to previous studies conducted in different parts of Ethiopia namely, Burie District 20.7% [56], Dabat 10.3 [19], Dello-Mena 14.3% [57], Lode Hetosa 15% [58], Kebribeyah Town, Somali Region, Eastern Ethiopia 12.3% [58], Dubti town 30.1% [59] and 2016 EDHS secondary data analysis report 23% [60]. It was also higher than studies conducted in Uganda 28% [61], India 17% [62], Nepal 32.8% [63], Western Africa 9.45% and Central Africa 6.9% [38] and Burundi 20% [54]. The difference might be attributed to cultural background of the populations, relatively high unmet need for family planning and reproductive characteristic variation [64]. Therefore addressing policy gaps, cultural and social context, unmet family planning needs, and individual reproductive characteristics can lead to better maternal health care utilization [48].

This study showed that socio-demographic, maternal and reproductive characteristics and knowledge level of the mothers were significantly associated with the utilization of modern PPFP methods. Accordingly, odds of using modern contraceptive during postpartum period among mothers of menses resumed were 1.63 more likely than counterpart. This agrees with the study done in Gondar town, South Gondar, East Gojam Zone, Northwest Ethiopia, Debre Berhan Town, Aroressa District, Southern Ethiopia and Malawi [16, 22, 25, 30, 32, 41, 65]. This might be due to postpartum women whose menses have returned after delivery may assume that they are at risk of getting pregnant, so this can encourage them to start postpartum FP methods early [66]. The other probable reason might be those women whose menses have resumed, and at the same time, their sexual activities may resume. Because of this, they may perceive that they are at risk of unintended pregnancy. The qualitative finding also supports this idea, which showed that resumption of menses encourages women to utilize postpartum contraceptive. Therefore, addressing socio-demographic factors, promoting education, enhancing partner communication, and providing targeted counseling can contribute to increased utilization of modern FP methods during the postpartum period [31].

This findings also showed that mothers who are linked to the FP unit during their child`s immunization were 1.96 times more likely to use modern contraceptive methods during postpartum period. This is in line with studies conducted in Injibara town, Northwest and Southern Ethiopia [31, 32, 46]. The possible explanation might be that child immunization creates a good opportunity for counseling about the advantage of FP utilization, birth space, and rising maternal and child health-related issues. Therefore to ensure these, maternal health care services and regular immunization services are a continuous point of contact to provide information about FP, offer services, and link women to PPFP services [67]. The qualitative finding also supports this idea, which showed that opportunity of counseling about the advantage of FP encourages women to utilize postpartum contraceptive. Therefore, integrating FP services with childhood immunization can improve contraceptive uptake and overall maternal and child health outcomes [68].

The odds of using modern contraceptive methods during postpartum period among women counselled about family planning during antenatal care visit were 1.63 more likely to utilize postpartum contraceptive than their counterpart. This finding is in line with studies conducted in Northern Ethiopia, Somalia Region, Eastern Ethiopia, North west Ethiopia and Aroressa District, Southern Ethiopia [8, 28, 32, 65] which indicated that mother counselled about family planning during antenatal care were more utilize contraceptive than counterpart. This might be due to those women who utilize family planning methods may be properly counseled by health care providers during their ANC visits about the available methods of FP and the consequences of frequent childbirths [69]. The qualitative finding also supports this idea, which showed that counselled women about the FP in the health facilities encouraged using postpartum contraceptive. Therefore, addressing misconceptions and fears related to contraceptive methods during ANC visits can help reduce unmet need for contraception. Integrating family planning services within ANC facilities can improve access and utilization. When women receive counseling during pregnancy, they are more likely to continue using contraception postpartum [70].

Mothers who have good knowledge towards modern contraceptive were 1.53 more likely to use modern contraceptive method during postpartum period. This finding is similar with studies conducted in Gondar Town, Injibara town, South Gondar, Butajira and Gozamen district [25, 30, 31, 41, 46]. The possible reason might be having good knowledge of FP methods may increase the chance of utilizing modern PPFP methods and suggested that time of starting postpartum family encourage mothers to initiate postpartum contraceptive utilization within one year after delivery [31, 71, 72]. Therefore, enhancing knowledge about modern contraceptive methods among mothers can lead to better family planning practices and contribute to maternal and child health [73].

Findings from the qualitative data summarized that, husband/partner oppose, myths and misconception on contraceptive, need for excess family size, religious prohibition, fear of side effect, menses not resumed, lack of counselling and privacy room, lack of accessibility to contraceptive methods and lack of transportation to health facility were barriers to use modern postpartum family planning. This findigs is similar with a facility-based cross-sectional study conducted in Western Ethiopia, which explored the barriers and determinants of postpartum family planning (PPFP) uptake among women visiting Maternal, Neonatal, and Child Health (MNCH) services in public health facilities [74]. This findings also similar with study conducted in zanzibar which stated that limited access to services, fear of side effects, cultural or religious opposition were barriers to modern contraceptive utilizations. To address these issues, increased knowledge about family planning methods and involving husbands in education and counseling during pregnancy, childbirth, and the postpartum period are crucial steps.

The study was used primary data and is community-based mixed methods which would help to know the real practice and dig out barriers that hinder the utilization of PPFP at the community level. However, social desirability bias might be the challenge when a woman answers the questions and women were asked sexual and reproductive behavior might diminish honest responses due to the cultural sensitiveness of the issue.

## Conclusion and recommendation

In this study, the prevalence of utilization of modern postpartum family planning methods during the postpartum period among postpartum women in konso district was low compared to the WHO recommendation for postpartum women. Menses resumption, link to the family planning unit during their child`s immunization, family planning counselling during antenatal care visit and good knowledge towards modern contraceptive were found to be significantly associated with postpartum contraceptive utilization. The qualitative, data also identified need for excess family size, religious prohibition, fear of side effect, menses not resumed, lack of counselling and privacy room, lack of accessibility to contraceptive methods and lack of transportation to health facility were barriers to use modern postpartum contraceptive utilization.

Therefore, enhancing family planning (FP) counseling during antenatal care (ANC) visits, delivery, and child immunization is crucial to minimize missed opportunities for postpartum women to access contraceptive methods. These interactions with healthcare providers serve as effective entry points for providing accurate information about FP options and reducing missed chances for postpartum women to adopt modern contraceptive methods. Additionally, empowering women through increased educational access plays a vital role in enhancing their knowledge about modern contraceptive utilizations at healthcare facilities.

Its also important to strengthen community-based education and counseling programs to address misconceptions and provide accurate information, promote awareness about contraceptive options through religious leaders and community influencers and improve access to contraceptive methods by expanding distribution points and ensuring transportation options for individuals seeking postpartum care. Encouraging bilateral discussions between women and their husbands about reproductive matters is essential also essential points. Furthermore, promoting the early use of modern FP methods before the resumption of menses can help mitigate the risk of unintended pregnancies.

## Data Availability

Data for this article is avaliable up on reasonable request for principal investigator.

## Abbreviations

ANC: Antenatal care
CI: Confidence interval
CSA: Census Statistical Agency
IDI: In-depth Interview
EDHS: Ethiopian Demographic Health Survey
HEW: Health Extension Worker
HW: Health Worker
IDI: In-Depth Interview
KII: Key Informant Interview
SNNP: Southern Nation and Nationalities People
WHO: World Health Organization

## Oral informed consents/patient consents

Each study participant was adequately informed by their data collectors about the purpose, contents of the study, method and anticipated benefit and risk of the study and the right to withdraw from the study at any time. An oral informed consent was obtained from the study participants’ before data collection during study time. In addition, participants were assured about the aim, possible risk if any, and confidentiality of the study using their own local language.
